# Post-treatment duration of positivity for standard and ultra-sensitive *Plasmodium falciparum* antigen-based rapid diagnostic tests, a cohort study from a low-endemic setting in Namibia

**DOI:** 10.1016/j.ebiom.2024.105489

**Published:** 2024-12-09

**Authors:** Henry Ntuku, Brooke Whittemore, Lucille Dausab, Ihn Kyung Jang, Allison Golden, William Sheahan, Xue Wu, Hannah Slater, Gonzalo J. Domingo, Smita Das, Elias Duarte, Lydia Eloff, Teun Bousema, Kjerstin Lanke, Cara Smith Gueye, Lisa M. Prach, Jaishree Raman, Petrina Uusiku, Stark Katokele, Roly Gosling, Bryan Greenhouse, Davis Mumbengegwi, Michelle S. Hsiang

**Affiliations:** a Malaria Elimination Initiative, Global Health Group, University of California, San Francisco (UCSF), San Francisco 94158, USA; b Division of HIV, Infectious Diseases, and Global Medicine, Department of Medicine, UCSF, San Francisco 94158, USA; c Department of Pediatrics, University of Texas Southwestern Medical Center, Dallas 75390, USA; d Multidisciplinary Research Centre, University of Namibia, Windhoek 13301, Namibia; e Diagnostics Program, PATH, Seattle 98121, USA; f Radboud University Medical Centre, Nijmegen 6500HB, the Netherlands; g Centre for Emerging Zoonotic and Parasitic Diseases, National Institute for Communicable Diseases, A Division of the National Health Laboratory Service, Sandringham, Johannesburg, Gauteng, South Africa; h Namibia Ministry of Health and Social Services, Windhoek 13198, Namibia; i Department of Pediatrics, UCSF Benioff Children’s Hospital, San Francisco 94158, USA; j Department of Epidemiology and Biostatistics, UCSF, San Francisco 94158, USA

**Keywords:** Malaria, Histidine-rich protein, Persistence, Low transmission, Low-endemic, Malaria elimination

## Abstract

**Background:**

The standard malaria rapid diagnostic test (RDT) and newer ultra-sensitive RDT (uRDT) target *Plasmodium falciparum* histidine rich protein-2 (HRP2), which persists post-treatment. The duration of test positivity has not previously been studied in a low transmission setting.

**Methods:**

We conducted a longitudinal cohort study in a low transmission setting in Namibia. RDT-positive individuals identified through passive and active case detection were treated and followed weekly for testing by RDT and uRDT, HRP2 quantification, quantitative PCR (qPCR) of parasitemia, and quantitative reverse transcriptase PCR (RT-PCR) of gametocytemia, until RDT and uRDT were negative for two consecutive weeks. Determinants of persistent positivity were identified using Cox proportional hazards models.

**Findings:**

Among 137 participants with complete follow-up and no evidence of resurgence during follow-up, median duration of positivity was 42 days (range: 3−98 range) for RDT, compared to 67 days (range 12–105) for uRDT. In a sub-analysis of those with laboratory data before treatment (n = 60), drug resistance did not explain persistent positivity. Younger age (<15 years versus ≥15 years: aHR: 1.85, 95% CI 1.04−3.30, and 1.67, 95% CI 0.96−2.89, for RDT and uRDT, respectively), higher initial parasite density (highest versus lowest tertile: aHR 0.11, 95% CI 0.04−0.32 and 0.19, 95% CI 0.07−0.48 for RDT and uRDT, respectively), and persistent parasitemia (≥7 days versus reference of <7 days, aHR 0.39, 95% CI 0.20−0.76, and 0.40, 95% CI 0.21−0.76 for RDT and uRDT, respectively) were associated with longer duration of positivity.

**Interpretation:**

Duration of RDT/uRDT positivity was more than double compared to reports from higher endemic settings, potentially due to lower population immunity to clear parasite DNA and antigen. Prolonged duration of positivity compromises their use to detect current infection, but increased detection of recent infection can facilitate surveillance and inform elimination efforts.

**Funding:**

The project was funded by the 10.13039/100000865Bill and Melinda Gates Foundation (A128488 and INV1135840), Horchow Family Fund (5300375400), and Chan Zuckerberg Biohub.

**Research in context**

**Evidence before this study**

Standard malaria rapid diagnostic tests (RDTs) and newer ultra-sensitive RDTs (uRDTs) target *Plasmodium falciparum* histidine rich protein-2 (HRP2), which persists post-treatment. In low transmission settings, lower immunity may alter duration of positivity, and thus complicate case management, and community-based surveillance and elimination efforts.

On May 17, 2024, we conducted a PubMed search for original articles with no restrictions on language or time period, using the search terms “histidine-rich protein 2 OR Rapid diagnostic test” AND “malaria” AND “persistence OR dynamics.” We found one systematic review of 31 studies that modelled the mean duration of HRP2 RDT positivity to be 15 days (95% CI 5–32); no studies were from low transmission settings. Subsequently, uRDTs became available and we found two additional studies from a high transmission setting. Median duration of positivity was 7–14 days (maximum 50 days) for RDT and 21–33 days (maximum 47 days) for uRDT. Duration of positivity reflected persistence of HRP2 antigenemia, was associated with younger age, and was not associated with persistent sexual-stage parasitemia; association with persistent asexual-stage parasitemia or drug resistance was not assessed.

**Added value of this study**

This study is the first study on the duration of positivity of RDT and uRDT from a low transmission setting and the first that comprehensively examines factors associated with duration of positivity. We conducted highly sensitive quantification of HRP2 and molecular testing for sexual-stages, asexual-stages, and drug resistance. Post-treatment, RDT and uRDT remained positive for approximately 3–9 weeks and 6−12 weeks, respectively. Similar to prior studies, younger age and higher initial parasite density were associated with longer duration of positivity. We additionally found that duration of positivity was associated with persistence of asexual stage parasitemia, but not drug resistance. Duration of RDT/uRDT positivity was more than double compared to reports from higher endemic settings, potentially due to lower population immunity to clear parasite DNA and antigen.

**Implications of all the available evidence**

The findings from this study, together with prior studies from higher transmission settings, suggest that duration of RDT/uRDT positivity is longer in lower transmission settings, potentially due to lower population immunity to clear parasite DNA and antigen. RDT/uRDT should not be relied upon to detect current infection for several weeks post-treatment. However, increased detection of recent infection can facilitate community-based surveillance and elimination efforts.

## Introduction

Confirmation of malaria by either microscopy or rapid diagnostic test (RDT) before initiating antimalarial treatment in patients with suspected malaria is recommended.^1^ Due to their ease-of-use, RDTs have been readily adopted in settings where high quality microscopy is difficult to maintain. Worldwide, more than 300 million RDTs are sold each year.^2^ Similar to rapid tests for other infections such as SARS-CoV-2,^3^ malaria RDTs are antigen-based that can remain positive after infection has cleared. False positive results among recently infected individuals make it difficult to diagnose new or recrudescent infection, and for surveillance, RDT-based results may lead to over-estimates of malaria and divert attention from other possible causes of febrile illnesses.^4^

Using microscopy as gold standard, early studies of RDTs demonstrated over 90% sensitivity and specificity for *P. falciparum* infection among patients presenting with fever.^5^^,^^6^ However, for malaria elimination strategies, more sensitive diagnostics may be needed. Community-based Test and Treat (TaT),^7^ or active case detection and treatment, is an elimination strategy that targets individuals with subclinical infection that may not present for care, but can perpetuate transmission. A positive RDT in a household, as indicator of recent exposure or ongoing transmission, has also been used as a trigger for presumptive treatment in an entire household.^7^ Numerous studies have shown that standard RDTs miss at least half and up to 90% of PCR-detectable infections.^7^ This challenge is of particular relevance to low-endemic settings, where compared to higher endemic settings, the relative proportion of infections with parasitemias below the detection limit of RDTs (about 100 parasites/μL) increases, presumably due to a high proportion of infections being chronic.^8^

An ultra-sensitive *P. falciparum*-specific RDT (uRDT) was developed to address the challenge of low density infections.^9^ Like standard *P. falciparum*-specific RDTs, the uRDT is an immune chromatography-based assay that detects histidine rich protein 2 (HRP2), an antigen that is known to persist in the bloodstream for 2−5 weeks post-treatment with artemisinin combination therapy (ACT).^10^ This persistence is related to splenic pitting, which is the main mechanism by which artemisinins clear parasite from infected red cells. HRP2 is exported into the cytosol of red cells, and after pitting, these red cells return to circulation.^11^ Models of HRP2 kinetics have shown that the duration of post treatment HRP2 positivity is a function of the limit of detection of an HRP2 test, hence, the uRDT, with a greater than 10-fold lower limit of detection for HRP2 compared to standard RDT, is likely to have longer duration of positivity.^9^

At the same time, recent years have seen the rise of infections with *P. falciparum* parasites for which the *hrp2* and *hrp3* genes have been deleted.^12^ In these parasites the HRP2 protein and the analogous and often cross-reactive protein HRP3 are no longer expressed resulting in false negative RDT results even in sick patients presenting with high parasite density infections.^13^ The prevalence of infections *hrp2/hrp3* deletions is very heterogenous even within a country leading to difficult decision making regarding when to adopt RDTs that detect lactate dehydrogenase (LDH), which until recently represent a drop in clinical sensitivity.^14^

To inform the use of next-generation ultra-sensitive RDT (uRDT) for diagnosis and surveillance, research regarding their duration of positivity is needed. Additionally, understanding the dynamics of HRP2 can inform the development of new RDTs that should perform with equal sensitivity regardless of the presence of *hrp2/hrp3* deletions. Here, we aimed to measure the duration of positivity for standard and ultra-sensitive HRP2-based RDTs following treatment for uncomplicated *P. falciparum* malaria infection, and identify factors associated with persistent positivity.

## Methods

### Study setting

The study was conducted in the Zambezi region, northern Namibia from May to September 2018. During the study, malaria transmission intensity was low: annual incidence of 40 cases per 1000 population in the year prior the study, and malaria prevalence of 0.8% detected by RDT and 2.2% detected by loop-mediated isothermal amplification (LAMP) in 2015.^15^ Malaria transmission is seasonal, with most cases occurring from January to June. Almost all malaria cases are due to *P. falciparum*. At the time of the study, the national malaria policy recommended parasitological confirmation by RDT at the health facility level and RDT or microscopy at the hospital level, and CareStart™ Malaria Pf/PAN (HRP2/*Plasmodium* lactate dehydrogenase or pLDH) Antigen (AccessBio) was the standard RDT used nationwide.

### Study design and sample size

This was a prospective longitudinal single arm cohort study. The sample size was calculated to estimate the time to become negative of HRP2-based RDTs. It was calculated that a minimum of 137 participants were needed to provide 10% precision with 95% confidence around the estimate of when 20% of participants were uRDT positive; 20% chosen as a reasonable threshold to inform treatment decision or intervention response. Accounting for standard RDTs lower positive predictive value (5% false positive) and 10% lost to follow up, the goal sample sizse for enrollment was 162 participants.

### Patient recruitment and follow up

Between May and June 2018, the study recruited consecutively RDT (CareStart™ Malaria RDT, AccessBio) positive patients over 6 months of age with uncomplicated *P. falciparum* malaria attending the regional hospital outpatient department or 12 health clinics within the hospital catchment area or detected during Ministry of Health and Social Services (MOHSS) screen and treat activities conducted in the community. Patients were invited to participate, screened for eligibility, and then enrolled if they met criteria, including provision of informed consent (see inclusion and exclusion criteria, Supplemental Table S1). At clinics where a higher number of malaria cases were anticipated, study staff enrolled participants at the health facility prior to administration of antimalarials. For malaria cases identified at other health facilities or in the community, study staff enrolled participants after treatment was started but within 7 days. At enrollment, capillary blood was collected for uRDT testing, HRP2 concentration, and molecular testing. A short questionnaire was completed to collect self-reported information regarding demographic characteristics, travel history, complete medical history (including prior and concomitant medication), and contact details. Household location was recorded.

Participants were treated per national guidelines with the ACT, artemether-lumefantrine (AL, Coartem®, Novartis, Switzerland, Komefan 140®, Mylan Laboratories, Netherlands) with or without the gametocytocidal drug, single low dose of (PQ, Remedica, Cyprus). The first dose of treatment was directly observed with a snack provided to maximise absorption. For subsequent doses, participants were instructed to take with a meal. Reminder phone calls were made for subsequent doses and adherence was assessed by observing the blister pack on day 3.

Participants were followed up at seven-day intervals or anytime if condition deteriorated or fever reoccurred. At each follow up visit, a clinical assessment was performed, and blood was collected for RDT and uRDT testing, as well as subsequent testing for HRP2 concentration and molecular testing. Follow up was discontinued when both RDTs were negative for two consecutive visits. Follow up visits were organised at the regional hospital and one of the health clinics within the hospital catchment area (Katima clinic) with compensation provided for transport. If at enrollment, the participant indicated inability to visit one of the follow-up sites, or if the participant did not attend a scheduled follow up visit, active follow-up visit in the community was performed. Participants who did not attend two consecutive follow up visits and could not be found during active follow up were considered lost to follow up. If during the course of the study a subject required clinical care, they were referred to the regional hospital or one of the health clinics.

### Laboratory methods

RDT (CareStart™ Malaria Pf HRP2/PAN pLDH) and uRDT (Alere™ Malaria Ag Pf) testing was conducted per manufacturer’s instructions and by trained nurses. From a finger prick, 250 μL of whole blood was collected in a BD Microtainer® tube with Ethylenediaminetetraacetic acid (EDTA) additive, transferred into and stored at −80 °C in separate cryotubes for subsequent HRP2 enzyme-linked immunosorbent assay (ELISA), deoxyribonucleic acid (DNA) polymerase chain reaction (PCR), using packed red blood cells after whole blood was centrifuged, and quantitative reverse transcriptase real-time PCR (qRT-PCR) using whole blood mixed with RNAprotect (Qiagen) in a 1:5 ratio. HRP2 ELISA was conducted on all samples. DNA PCR and qRT-PCR were conducted on first time point samples, with testing of subsequent timepoints until the participant was negative.

HRP2 concentration in whole blood was determined by ELISA using the Q-plex Human Malaria Array (Quansys Biosciences).^16^ Samples were considered positive if HRP2 concentration was greater than 2.3 pg/mL. For DNA PCR, whole blood was centrifuged, and packed red blood cells were stored at −20 °C before undergoing DNA extraction using the Quick-DNA miniprep kit (Zymo Research Corp, Irvine, CA, USA) and a qPCR targeting the varATS region^17^ using template DNA corresponding to 10 μL of whole blood. Parasite density was estimated by averaging duplicate runs and samples were considered positive if parasite density was greater than 0.1 parasites/μL. To identify non-falciparum species, PCR targeting the *Plasmodium* mitochondrial cytochrome *c* oxidase III (cox3) gene was performed, as previously described,^18^ on all samples positive by qPCR, *Plasmodium* LDH or *Plasmodium vivax* LDH (available from the Q-plex), or with an LDH positive line on the RDT. The only deviation from the previously described protocol is that the first PCR product was diluted 1:10 instead of 1:50. For gametocyte stage detection, nucleic acid was extracted using a MagNA Pure LC automatic extractor (Roche Applied Science), followed by qRT-PCR targeting Pfs25 and SBP-1 mRNA to detect female gametocyte and ring stage parasites, respectively.^19^^,^^20^

To investigate whether persistently positive RDT and uRDT results were potentially due to new infection or persistent infection, amplicon deep-sequencing targeting the 236 base-pair segment of apical membrane antigen 1 (AMA-1) was conducted using methods previously described to distinguish haplotypes.^21^ All samples from individuals that had PCR positivity beyond the median uRDT duration of positivity were sequenced in replicate. For each time point, complexity of infection was measured and the presence or absence of each haplotype was ascertained. To investigate whether treatment failure may have explained persistent infection, we used previously described methods to amplify the propeller domain *of Pfkelch13* gene and reviewed sequences for known polymorphisms associated with delayed parasite clearance.^22^ This was conducted among cases that were classified as recurrence and sufficient sample was remaining to conduct testing.

### Statistics

Data were collected using password protected tablets equipped with Open Data Kit software (ODK, University of Washington & Google Foundation) and analyzed using Stata 14 software (Stata Corporation College Station, TX, USA). Baseline clinical and epidemiological characteristics were summarised. Dichotomous variables were summarised as percentages with 95% confidence intervals (95% CI). Continuous variables were described using their mean and standard deviation (SD), or median and range if the distribution was skewed. Laboratory results were stratified by those enrolled pre-treatment versus those that were not. The primary outcome was the duration of positivity of standard RDT and ultra-sensitive RDT, which was defined as the time between initiation of treatment and the first day the test is reported negative for two consecutive weeks. The probability of the test remaining positive over time was estimated using the Kaplan–Meier survival function, and the log rank test was used to compare survival rates. Follow-up started with day of treatment and ended with day that the results for both RDT and uRDT were negative for two consecutive visits. Participants were censored if they withdrew, were lost to follow-up, or had possible resurgence of infection as observed by rise in *Plasmodium* LDH during follow-up or clinical assessment (symptoms and new or persistent RDT positivity). Kaplan–Meier curves were visually reviewed to check if the Cox model proportional hazard assumption - that the relative hazard remains constant over time for different predictor levels - was not violated.

For participants with blood collected prior to treatment, a sub-analysis was conducted using univariate Cox proportional hazards regression models to estimate the association between epidemiological factors (self-reported sex, age, detection at health facility or in community screen and treat), laboratory values (parasite density, HRP2 concentration, and presence of gametocytes at diagnosis, as well as persistence of positivity by qPCR) and the RDT or uRDT duration of positivity. Start of follow-up, end of follow-up, and censoring were as per the Kaplan–Meier analysis. A Directed Acyclic Graph (DAG) was generated to identify consider causal pathways and identify confounding variables that required conditioning in adjusted models. Models were specified to examine a specific factor–outcome association (Supplemental Fig. S1 and Table S2). Finally, to visualise decay of parasite densities and HRP2 concentrations, boxplots showing median and range of value at follow-up visits were generated.

### Ethics

The study protocol was approved by the Research Unit of the MOHSS of Namibia (Ref: NH), the University of Namibia Research Ethics Committee (MRC/382/2018) and the University of California, San Francisco Committee for Human Research (17-24178). Written informed consent was obtained from all participating adults and from parents or guardians of children, and minor assent was obtained from minors 12–17 years of age.

### Role of funders

The funders contributed to, but did not lead, the study design. The funders had no role in data collection, data analysis, interpretation, or writing of the manuscript.

## Results

### Participants characteristics

**Fig. 1:**
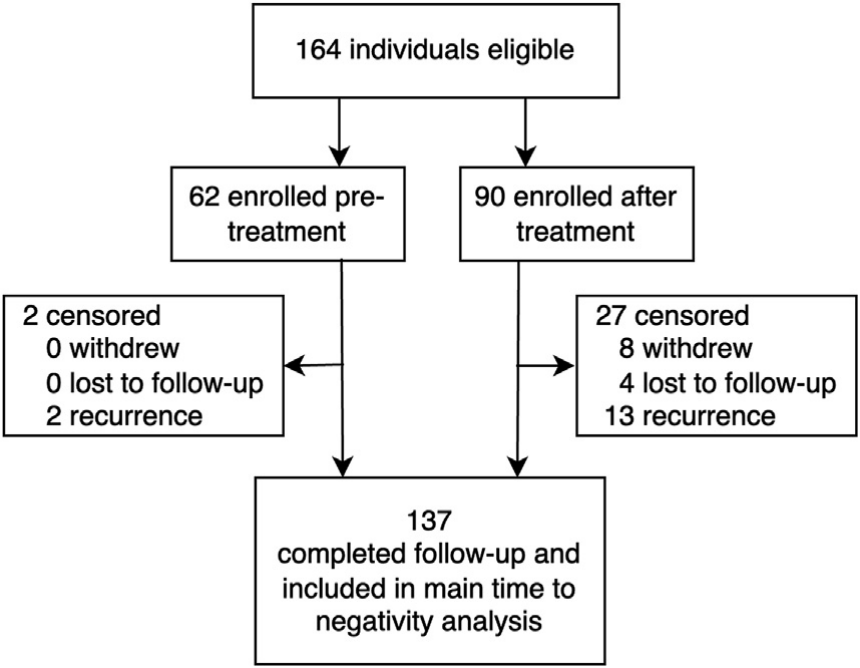
Enrollment and follow-up flowchart.

Of 164 total participants enrolled, 12 withdrew or were lost to follow-up and 15 were classified as resurgent based on clinical assessment (n = 5) or rise in LDH (n = 10) during follow-up. Of 137 participants included in the longitudinal analyses, 60 were enrolled on Day 0, enabling blood collection prior to treatment and inclusion in a sub-analysis of factors associated with duration of positivity (Fig. 1). Overall, enrollment occurred at a median of 1 day (range 0–19) after treatment. Baseline demographic and clinical characteristics of participants are shown in Table 1. 55.3% of participants were male and 56.6% were 15 years or older. Most participants were recruited from health facilities (83.6%), including all Day 0 enrollees, versus during community screen and treat activities. Among participants with blood collected before treatment, at enrollment, median HRP2 concentration was 1,387,566 pg/mL (range 1.007−1.09e+10), median parasite density was 4309 parasites/μL (range 0.2–131,942), and 78.7% were gametocyte positive by qRT-PCR, with a mean density of 379 gametocytes/μL (range 10–84,900). Compared to participants enrolled pre-treatment, symptomatic participants recruited at health facilities had similar levels of qPCR positivity, parasite density, and gametocytemia, though HRP2 concentration at baseline was nearly double (median 2,483,233 versus 1,397,566 pg/mL).

**Table 1:** Baseline characteristics of study participants.

Participant characteristics at enrollment	N = 152
Age (years), median (range)	17 (1–80)
Age category, n (%)	
<5 years	14 (9.2)
5–14 years	52 (34.2)
≥15 years	86 (56.6)
Sex, n (%)	
Male	84 (55.3)
Female	68 (44.7)
Recruitment method, n (%)	
Health facility	127 (83.6)
Community test and treat^a^	25 (16.5)
Standard RDT result, n (%)	
*P. falciparum* (HRP-2 positive only)	65 (42.8%)
*P. falciparum* or mixed infection (HRP-2 and Pan LDH positive)	84 (55.3%)
Enrolled pre-treatment, n (%)	
Yes	62 (40.8)
No	90 (59.2)
Day since treatment, median (range) (N = 90)	4 (1–19)
HRP2 concentration (pg/mL), median (range)	
All (N = 150)	399,397 (1−1.09e+10)
Enrolled pre-treatment (N = 61)	1,387,566 (1−1.09e+10)
Enrolled after treatment (N = 50)	2,483,233 (2205−1.09e+10)
qPCR positive, n (%)	
All (N = 149)	133 (89.3%)
Enrolled pre-treatment (N = 59)	55 (93.2%)
Enrolled after treatment (N = 48)	48 (100%)
Parasite density (parasites/μL), median (range)	
All (N = 149)	5.6 (0−131,942)
Enrolled pre-treatment (N = 55)	4309 (0.2−131,942)
Enrolled after treatment (N = 48)	4613 (0.2−131,942)
Gametocyte positive, n (%)	
All (N = 152)	68 (46.0%)
Enrolled pre-treatment (N = 61)	48 (78.7%)
Enrolled after treatment (N = 43)	43 (86%0.0)
Gametocyte density (gametocytes/μL), median (range)	
All (N = 68)	362 (3−84,900)
Enrolled pre-treatment (N = 48)	379 (10−84,900)
Enrolled after treatment (N = 43)	381 (15−84,900)

HRP2 histidine-rich protein 2.

a 24 participants were afebrile and 1 was febrile at presentation.

### RDT and uRDT duration of positivity

**Fig. 2:**
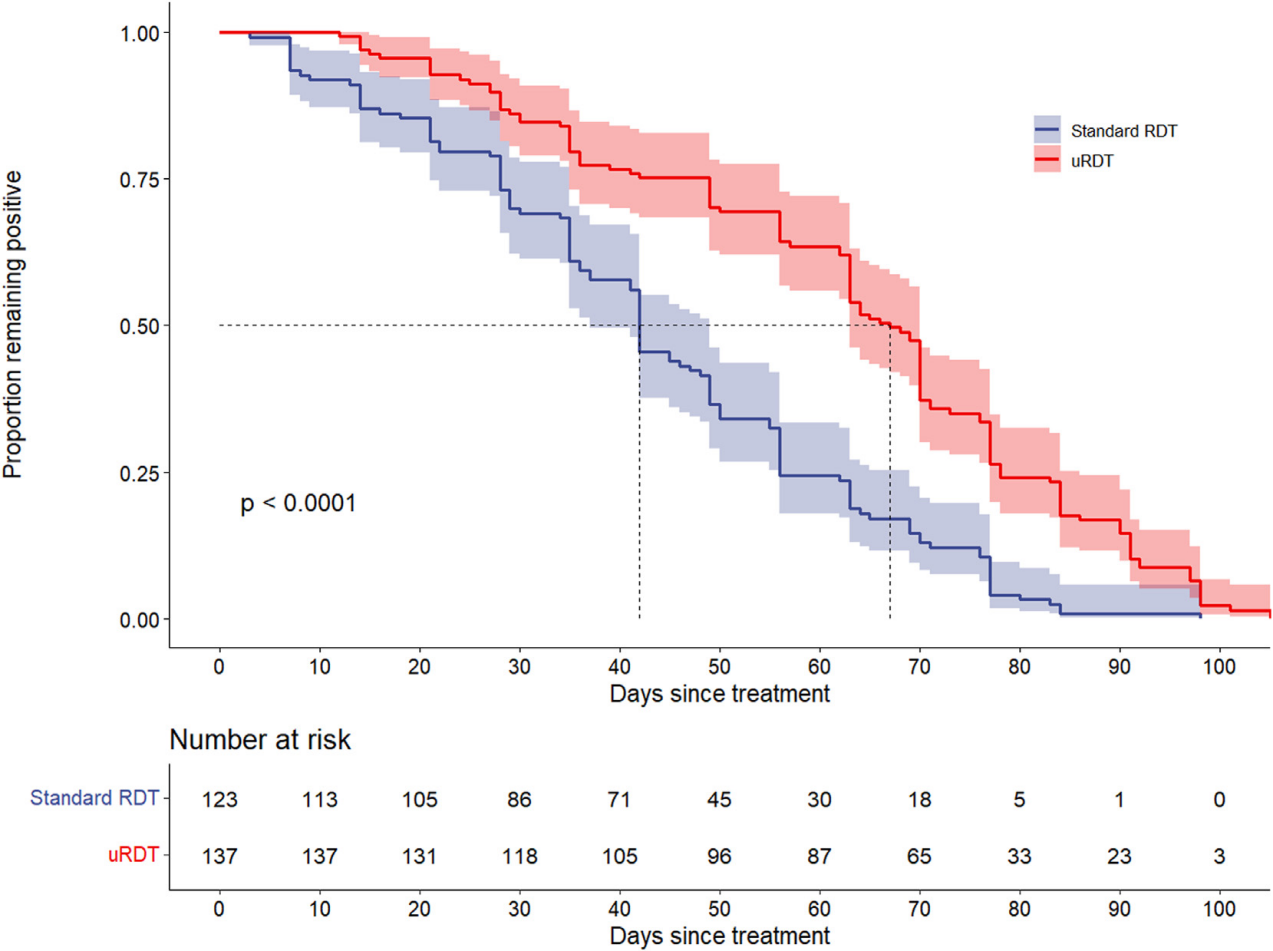
Kaplan–Meier curves of proportion remaining RDT or uRDT positive by days since treatment, n = 137 participants.

Participants were followed up to a maximum of 132 days after treatment. With the exception of censored participants, all became negative by standard RDT and uRDT by the end of the follow-up period. Median duration of positivity of standard RDT and uRDT was 42 days (IQR: 28−56, range: 3−98 range) and 67 days (IQR: 49−78, range 12–105), respectively (Fig. 2). As the study was powered to assess timepoint when 20% ± 10% remained positive, we identified these timepoints. The time at which 20% (95% CI 14%–28.2%) were still positive was 76 days by RDT and 84 days by uRDT.

### HRP2 concentration and parasite density at enrollment and follow-up

**Fig. 3:**
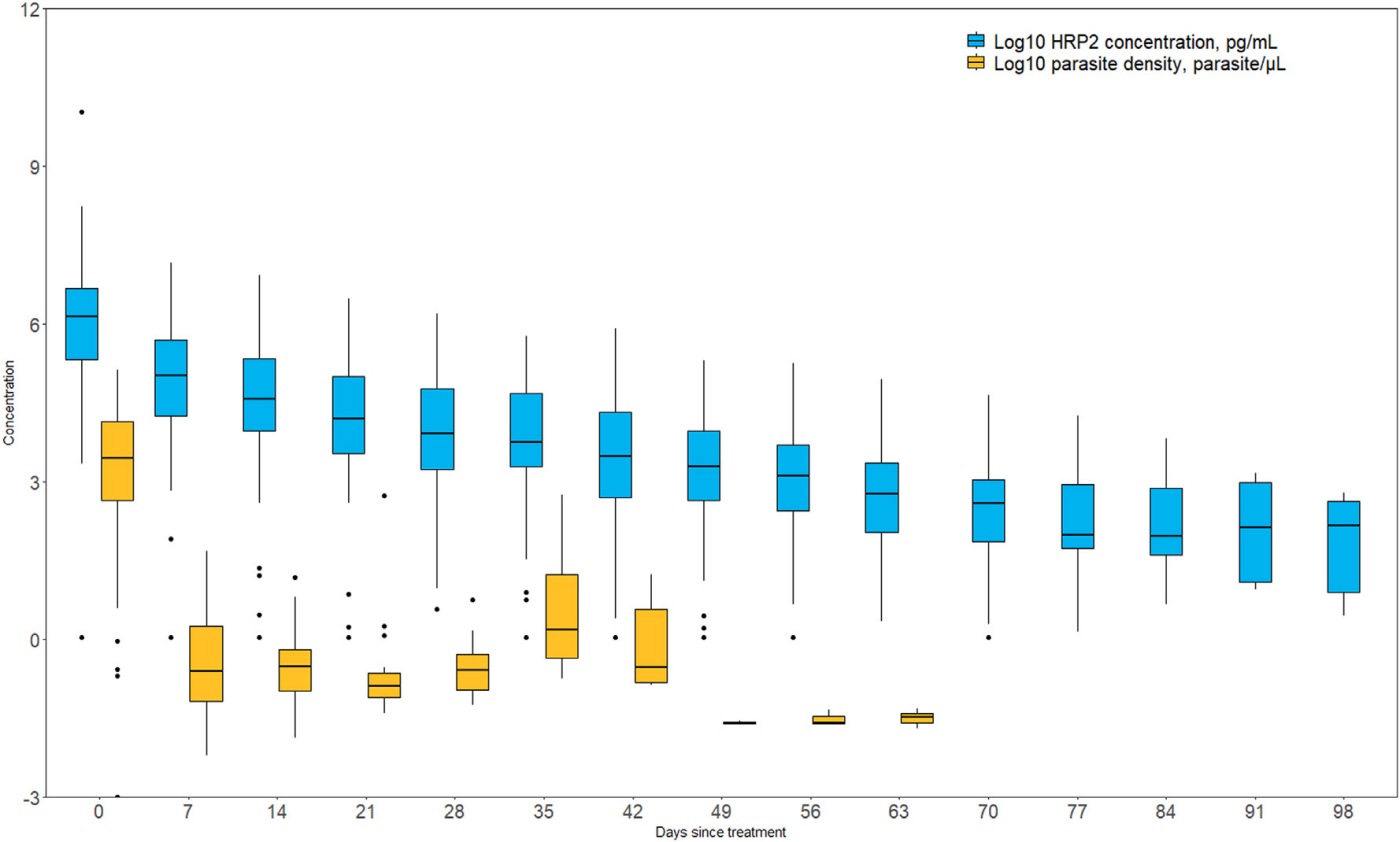
Boxplots of log10 HRP2 concentration and parasite density by days since treatment, median, interquartile range, minimum and maximum.

For the 137 participants included in the longitudinal analysis, HRP2 antigenemia was detected up to 105 days after treatment. The median duration of positivity of HRP2 antigenemia was 70 days (95% CI 14−98). Parasitemia by qPCR was detected up to day 98. The median duration of positivity of qPCR was 7 days (95% CI 5−42). Gametocyte and ring stages were detected until day 21, at which point sampling of subsequent samples was stopped due to few remaining positives. Median duration of female gametocyte and asexual ring-stage positivity was 2 days (range 2–18) and 10 days (range 5–20), respectively. For participants enrolled pre-treatment only, HRP2 concentrations and parasite densities measured at weekly follow-up are shown in Fig. 3.

### Factors associated with duration of test positivity

**Fig. 4:**
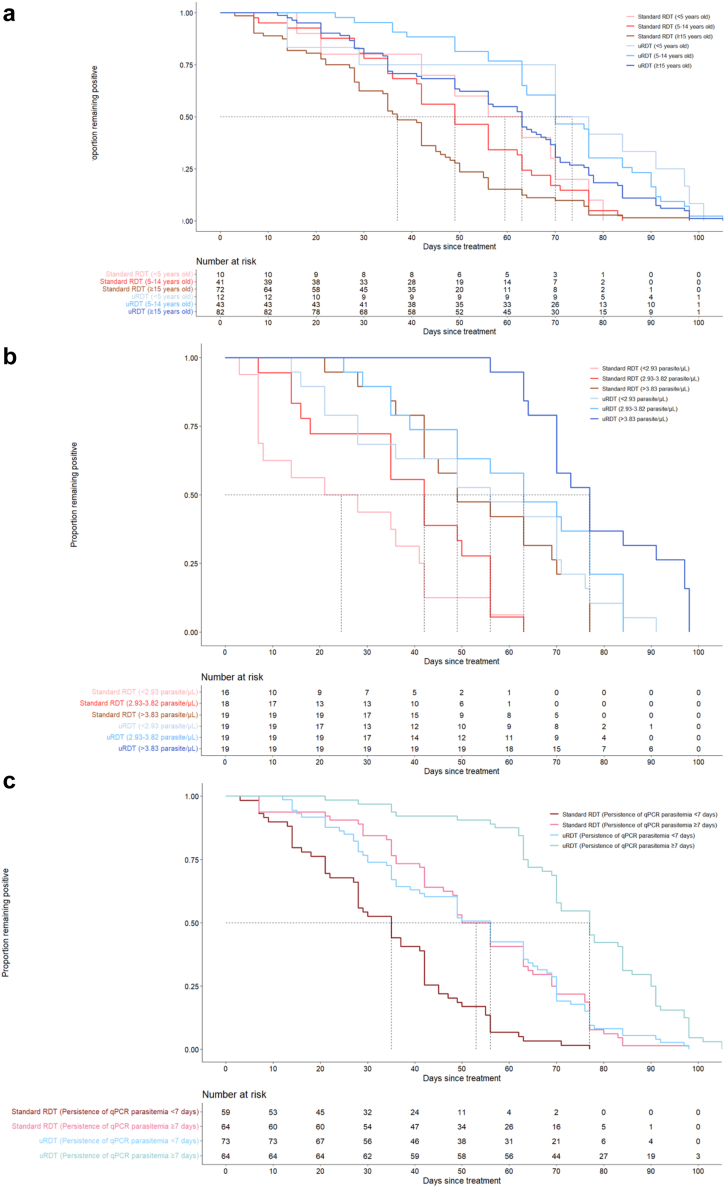
Kaplan–Meier curves of proportion remaining RDT or uRDT positive by days since treatment, stratified by age category (a), initial parasite density (log10 parasites/μL) (b), and persistence of positivity by qPCR (c), n = 137 participants.

Unadjusted and adjusted HR examining potential relationships between epidemiological and clinical factors with duration of positivity for standard RDT and uRDT are shown in Table 2. For each covariate of interest, Kaplan–Meier curves were visually observed to be roughly parallel, thus in adherence with the Cox proportional hazards assumption. Individuals 15 years and older had shorter duration of positivity compared to those less than 15 years, particularly with the RDT (RDT: aHR 1.85, 95% CI 1.04−3.30, uRDT: 1.67, 95% CI 0.96−2.89, respectively; also shown in Supplemental Fig. S2a). There were too few participants <5 years (n = 7) to include this age category in the multivariable model but Kaplan–Meier survival curves of RDT and uRDT positivity stratified by age <5, 5−14, and ≥15 years show a consistent trend in the association between younger age and longer duration of positivity (Fig. 4a). For age <5, 5−14, and ≥15 years, median duration of RDT positivity was 60 days (95% CI 42−70), 49 days (95% CI 42−56), and 37 days (95% CI 34−42), respectively, and for uRDT positivity, it was 74 days (95% CI 50−94), 70 days (95% CI 64−77), and 63 days (95% CI 56−68), respectively.

**Table 2:** Association of time to rapid diagnostic test negativity with demographic, clinical, and laboratory factors, among Day 0 enrollees.

	N = 60^a^ n (%)	Standard RDT time to negativity				uRDT time to negativity			
		HR (95% CI)	p-value	aHR^b^ (95% CI)	p-value	HR (95% CI)	p-value	aHR^b^ (95% CI)	p-value
Age group									
<15 years	26 (43.3)	Reference	0.060	Reference	0.036	Reference	0.096	Reference	0.067
≥15 years	34 (56.7)	1.67 (0.98–2.85)		1.85 (1.04–3.30)		1.55 (0.93–2.59)		1.67 (0.96–2.89)	
Sex									
Male	37 (61.7)	Reference	0.720	Reference	0.297	Reference	0.794	Reference	0.401
Female	23 (38.3)	1.10 (0.64–1.90)		1.36 (0.76–2.44)		1.07 (0.64–1.81)		1.27 (0.73–2.21)	
Recruitment method									
Health facility	50 (83.3)	Reference	0.111	Reference	0.141	Reference	0.199	Reference	0.211
Community test and treat	10 (16.7)	1.81 (0.87–3.76)		1.73 (2.83–3.60)		1.57 (0.79–3.13)		1.55 (0.78–3.10)	
Presence of gametocytes at enrollment									
No	13 (22.0)	Reference	0.244	Reference	0.111	Reference	0.377	Reference	0.107
Yes	46 (78.0)	0.68 (0.35–1.31)		2.34 (0.82–6.66)		0.75 (0.40–1.41)		2.28 (0.84–6.21)	
qPCR parasite density at enrollment log10 (parasites/uL)									
<2.93	19 (33.3)	Reference		Reference		Reference		Reference	
2.93–3.82	19 (33.3)	0.60 (0.30–1.18)	0.135	0.35 (0.14–0.86)	0.022	0.76 (0.40–1.44)	0.394	0.48 (0.21–1.10)	0.084
>3.83	19 (33.3)	0.23 (0.11–0.49)	<0.001	0.11 (0.04–0.32)	<0.001	0.32 (0.16–0.64)	0.001	0.19 (0.07–0.48)	<0.001
Persistence of qPCR parasitemia (days)									
Less than 7	28 (46.7)	Reference		Reference	0.005	Reference		Reference	0.005
≥7	32 (53.3)	0.30 (0.16–0.56)	<0.001	0.39 (0.20–0.76)		0.34 (0.19–0.61)	<0.001	0.40 (0.21–0.76)	
HRP2 concentration at enrollment log10 [(pg/mL)]									
<5.45	20 (33.3)	Reference		Reference		Reference		Reference	
5.45–6.538	20 (33.3)	0.45 (0.23–0.88)	0.019	0.75 (0.33–1.70)	0.494	0.45 (0.23–0.87)	0.018	0.62 (0.27–1.39)	0.245
≥6.54	20 (33.3)	0.34 (0.17–0.68)	<0.001	0.62 (0.24–1.58)	0.314	0.32 (0.16–0.63)	0.001	0.42 (0.17–1.06)	0.066

HR = hazard ratio; aHR = adjusted hazard ratio.

a 60 participants included, for which blood was collected prior to treatment and lab results were complete.

b Multivariate models with adjustments per Supplemental Table S2.

Higher initial parasite density and persistence of qPCR-detectable parasitemia (≥7 days versus reference of <7 days) were associated with longer RDT and uRDT duration of positivity (Table 2, Fig. 4b and c). A dose-dependent relationship was seen between higher parasite density and duration of test positivity. When qPCR-detectable positivity was ≥7 days for uRDT, 80% of participants remained positive at 63 days, versus 80% of participants remaining positive 28 day for when qPCR-detectable positivity was <7 days.

While higher HRP2 concentration at baseline was associated with longer duration of positivity for uRDT and standard RDT in the unadjusted analysis, confidence intervals crossed 1.0 in the adjusted analysis. Kaplan-survival curves for uRDT and RDT positivity stratified by baseline HRP2 concentration (Supplemental Fig. S3). For both uRDT and standard RDTs there was no evidence of association between the duration of positivity and sex, detection through facility or in community test and treat, or baseline gametocytemia.

### Evaluation for new infection or drug resistance as potential cause of resurgence

Among participants that had uRDT positivity beyond the median duration of 67 days (n = 3) sequencing was conducted on longitudinal samples. Based on new haplotypes after Day 0, persistence of positivity was likely due to new infection (Supplemental Fig. S4), and this was consistent with these infections being classified as resurgent based on clinical assessment. Among all 15 participants with resurgence, six had follow-up samples available to conduct sequencing of the *Pfkelch13* gene. Samples from five of these participants showed wild type and Pfkelch P441L was identified in one sample.

## Discussion

In the last two decades, the scale-up of RDT has enabled a shift away from empiric treatment, and improved surveillance data from health facilities. In the last decade, renewed interest in malaria elimination has stimulated interest in the development of more sensitive diagnostics to detect lower level infections which can cause illness, and perpetuate transmission. The uRDT is one such tool, though its reliance on detection of HRP2 antigen, which persists after treatment, may compromise its utility in differentiating active infection versus a recent prior infection. Here, we provide new evidence on the duration of positivity of uRDT compared to standard RDT in a low transmission setting. Post-treatment, uRDT remained positive for approximately 6–12 weeks, compared to 3−9 weeks to standard RDT. Younger age and persistence of qPCR-positivity beyond one week were associated with longer duration of positivity. Prolonged duration RDT/uRDT positivity compromises their use to detect current infection post-treatment, but can facilitate surveillance to detect recent infection in elimination settings.

In this study, the duration of positivity of standard RDTs (median 42 days, maximum 98 days) is longer than what has been previously reported. In a systematic review and meta-analysis on the persistence of RDT positivity post-treatment in 31 publications, the modelled mean duration of positivity was 7 days (95% CI 2–20) for RDTs that detect both HRP2 and pLDH, 15 days (95% CI 5–32) for HRP2 RDTs.^23^ The maximum time to negativity was not reported as follow-up due to paucity of follow-up data beyond 42 days. The higher duration of positivity in our study may have been explained by recent improvement in the quality of standard RDTs. However more recently in a treatment efficacy trial from Mali, it was found that the median duration test positivity after treatment with ACT or ACT with single low dose of primaquine for RDT was 7 and 14 days, respectively (maximum 42 days for either treatment), and for uRDT was 21 and 28 days, respectively (maximum 50 days).^24^ In longitudinal follow-up from a vaccine trial in Mali, uRDT was positive for median 33 days (95% CI 28−47 days) after treatment.^25^ To our knowledge, these are the only other studies to report on the duration of positivity for the uRDT. The shorter durations of positivity reported in Mali, compared to a median of 67 days (maximum 105) in our study, may be due to pre-existing natural and/or vaccine-induced immunity in a high transmission setting. The longer duration of RDT/uRDT positivity in our study reflects a longer duration of HRP2 antigenemia: 70 days (maximum 98 days), versus 14 days (maximum 49 days) in the Mali study. Consistent with our finding that initial HRP2 concentrations were not associated with duration of positivity, the HRP2 concentrations in our study (6 log, pg/mL) were not higher than in the Mali studies (8 log and 4 log in the treatment efficacy and vaccine trials, respectively).

Consistent with other studies, we found longer duration of RDT/uRDT positivity to be associated with higher initial parasite density.^24^^,^^26^ Prior studies did not consider persistence of parasitemia, for which we found strong associations with duration of test positivity, even after adjusting for initial parasite density.^27^

Others have also documented that high proportion of patients having PCR-detectable parasitemia persisting for several weeks after treatment and clearance of microscopy-detectable infection.^28^ We considered potential factors contributing to the persistence of parasitemia and antigenemia, including treatment efficacy, gametocytemia, prior duration of infection, sex, age, and immunity.^29^ Intrinsic drug resistance was unlikely to have been a major contributor given the absence of kelch13 artemisinin resistance markers among most samples classified as recurrent. Also, in a separate sub-analysis of treatment efficacy (Supplemental Tables S3–S5) where we used a cutoff of parasite density <100 parasites/μL as a proxy for microscopy positivity, there we found no cases of early treatment failure or inadequate clinical or parasitological failure.^30^ Although we did not conduct directly observed therapy, lack of adherence was unlikely based on pill count conducted on the day 3 follow-up. Pharmacokinetic factors or in-vivo HRP2 dynamics^31^ were not examined but may have played a role. The presence of gametocytes has been proposed as a cause of persistent parasitemia and HRP2 positivity though consistent with another study, we did not find this association.^24^ In an area previously with high transmission intensity, males were found to clear parasitemia at a slower than females^21^; we did not find sex-based differences. Lower levels of acquired immunity in our lower transmission setting could have contributed to the delayed clearance of parasites and/or HRP2 in previously infected red cells. That younger age was associated with longer duration of positivity is also consistent with this hypothesis. While we did not explore this, lower complexity and diversity of infection may have also contributed to persistence of parasitemia in this low transmission setting.^32^

A weakness of our study was that we did not have sufficient staff to cover all facilities and track the number of individuals failing screening or refusing to participate. However, the demographics of study participants (age and sex) were similar to malaria case surveillance data from a coincident study, suggesting selection bias was likely minimal.^33^ Recruitment of some participants through active case detection also enabled inclusion of participants that may have otherwise been missed through standard passive case detection.

In conclusion, in this longitudinal cohort study, we found prolonged post-treatment duration of uRDT positivity compared to standard RDT. In adjusted models taking into consideration causal pathways, age, initial parasite density, and persistence of parasitemia were associated with persistence. We also found prolonged duration of RDT positivity compared to previously reported, potentially related to lower levels of immunity in this low transmission setting. Prolonged duration RDT/uRDT positivity compromises their use to detect current infection post-treatment. To better diagnose current malaria, and prevent its over-diagnosis, microscopy should be conducted if RDT or uRDT was positive in the prior 100 days. Tests that additionally target more rapidly decaying antigens, e.g., lactate dehydrogenase (LDH),^23^ can address this challenge, and may become more commonly used over HRP2-only diagnostics as HRP-deleted strains become more prevalent.^12^^,^^34^ Nonetheless, for low transmission and elimination settings, the persistence of HRP2 is a feature that can still be exploited to facilitate surveillance of recent infection in individuals and the community.

## Contributors

MSH, RG conceptualised and designed the study. BG, DM, HN, TB, and GD contributed to study design. HN and DM led the field implementation and supervision. BW, CSG, LP, and LD supported the field implementation. PU and SK supported collaboration with Ministry of Health and Social Services. ED and KL conducted the molecular testing. IKJ, AG, and SD conducted the parasite specific antigen assays. BG, TB, and GD provided additional oversight of the laboratory activities. BW and XU supported data management. MKD, WS, and HS supported data analysis. HN, XW, and MSH conducted the data analysis. HN wrote the first draft and MH revised the manuscript. All authors supported data interpretation and approved the manuscript final draft.

## Data sharing statement

After publication, data collected from this study are available upon request to the corresponding author. Available data include de-identified individual participant data and a data dictionary defining each field in the set. Requests to conduct analyses outside the scope of this publication will be reviewed by the principal investigators (MSH and DM) to determine whether a requester’s proposed use of the data is scientifically and ethically appropriate and does not conflict with constraints or informed consent limitations identified by the institutions that granted ethical approval for the study. Requests to reanalyse the data presented in this Article will not require such review.

## Declaration of interests

All authors declare no competing interests.
