# Supplementary material for: Post-treatment duration of positivity for standard and ultra-sensitive Plasmodium falciparum antigen-based rapid diagnostic tests, a cohort study from a low-endemic setting in Namibia
Source: eBioMedicine. 2024 Dec 9;111:105489. doi: 10.1016/j.ebiom.2024.105489 (PMC11683224; doi:10.1016/j.ebiom.2024.105489)
Supplement: Supplemental Figs. S1–S4 and Tables S1–S5 [file mmc1.docx]

**Supplemental**

Supplemental Table 1. Inclusion and exclusion criteria

| Inclusion criteria | 1. Usual resident of the village or the hospital catchment area 2. Age greater than 6 months 3. Mono-infection with *P. falciparum* confirmed by CareStart RDT or positive blood smear (i.e. no mixed infection) 4. Able to swallow oral medication 5. Ability and willingness to comply with the study protocol for the duration of the study and to comply with the study visit schedule 6. Informed consent from the participant or a parent/guardian 7. Informed assent from any minor participant aged from 12 to 17 years 8. Consent for pregnancy testing from female of child-bearing age (defined as age > 12 years and sexually active) or from their parent or guardian if under the age of 18 years |
| --- | --- |
| Exclusion criteria | 1. Presence of general danger signs in children aged under12 years or signs of severe falciparum malaria according to WHO definitions (Appendix 1) 2. Presence of febrile conditions due to diseases other than malaria (e.g. measles, acute lower respiratory tract infection, severe diarrhea with dehydration) 3. Ineligible to receive AL    1. Weight <5kg    2. Regular medication, which may interfere with AL pharmacokinetics (i.e. metoprolol, imipramine, etc.)    3. History of hypersensitivity reactions or contraindications to AL    4. First trimester of pregnancy (12 weeks)    5. Unable to or unwilling to take pregnancy test for women of child-bearing age (defined as age > 12 years and sexually active) 4. Known underlying chronic or severe diseases (e.g. cardiac, renal and hepatic diseases, HIV/AIDS) is recommended by WHO as an exclusion criterion. For this study, these individuals will not be excluded from the study but may be evaluated separately in the analysis |
| Exclusion criteria for primaquine* | 1. Pregnancy 2. Age <1 year 3. Infants weighing less than 10 kilograms 4. Women in the first 12 months of breastfeeding 5. Prior allergic reaction to primaquine |

*Presence of these exclusion criteria does not exclude patient from the study

Supplemental figure 1.

Supplemental Table 2. Model outputs

Model a/b: Age, adjusted for by sex; Sex adjusted for by age

|  | N=60*  n (%) | Standard RDT time to negativity | | | | uRDT time to negativity | | | |
| --- | --- | --- | --- | --- | --- | --- | --- | --- | --- |
|  |  | HR (95% CI) | p-value | aHR** (95% CI) | p-value | HR (95% CI) | p-value | aHR** (95% CI) | p-value |
| Age group | | | | | | | | | |
| <15 years | 26 (43.3) | Reference | 0.060 | Reference | 0.036 | Reference | 0.096 | Reference | 0.067 |
| ≥15 years | 34 (56.7) | 1.67 (0.98-2.85) |  | 1.85 (1.04-3.30) |  | 1.55 (0.93-2.59) |  | 1.67 (0.96-2.89) |  |
| Sex | | | | | | | | | |
| Male | 37 (61.7) | Reference | 0.720 | Reference | 0.297 | Reference | 0.794 | Reference | 0.401 |
| Female | 23 (38.3) | 1.10 (0.64-1.90) |  | 1.36 (0.76-2.44) |  | 1.07 (0.64-1.81) |  | 1.27 (0.73-2.21) |  |

Model c: Health seeking, adjusted for by: age and sex

|  | N=60*  n (%) | Standard RDT time to negativity | | | | uRDT time to negativity | | | |
| --- | --- | --- | --- | --- | --- | --- | --- | --- | --- |
|  |  | HR (95% CI) | p-value | aHR** (95% CI) | p-value | HR (95% CI) | p-value | aHR** (95% CI) | p-value |
| Health seeking/Recruitment method | | | | | | | | | |
| Health Facility | 50 (83.3) | Reference | 0.111 | Reference | 0.141 | Reference | 0.199 | Reference | 0.211 |
| Community test and treat | 10 (16.7) | 1.81 (0.87-3.76) |  | 1.73 (2.83-3.60) |  | 1.57 (0.79-3.13) |  | 1.55 (0.78-3.10) |  |
| Age group | | | | | | | | | |
| <15 years | 26 (43.3) | Reference | 0.060 | Reference | 0.042 | Reference | 0.096 | Reference | 0.070 |
| ≥15 years | 34 (56.7) | 1.67 (0.98-2.85) |  | 1.84 (1.02-3.31) |  | 1.55 (0.93-2.59) |  | 1.67 (0.96-2.91) |  |
| Sex | | | | | | | | | |
| Male | 37 (61.7) | Reference | 0.720 | Reference | 0.302 | Reference | 0.794 | Reference | 0.386 |
| Female | 23 (38.3) | 1.10 (0.64-1.90) |  | 1.37 (0.76-2.47) |  | 1.07 (0.64-1.81) |  | 1.28 (0.73-2.24) |  |

Model d: Initial parasite density, adjusted for by: age, sex, health seeking*, gametocyte at enrollment

|  | N=60*  n (%) | Standard RDT time to negativity | | | | uRDT time to negativity | | | |
| --- | --- | --- | --- | --- | --- | --- | --- | --- | --- |
|  |  | HR (95% CI) | p-value | aHR** (95% CI) | p-value | HR (95% CI) | p-value | aHR** (95% CI) | p-value |
| Initial parasite density by qPCR, log10 (parasites/uL) | | | | | | | | | |
| < 2.93 | 19 (33.3) | Reference |  | Reference |  | Reference |  | Reference |  |
| 2.93-3.82 | 19 (33.3) | 0.60 (0.30-1.18) | 0.135 | 0.35 (0.14-0.86) | 0.022 | 0.76 (0.40-1.44) | 0.394 | 0.48 (0.21-1.10) | 0.084 |
| >3.83 | 19 (33.3) | 0.23 (0.11-0.49) | <0.001 | 0.11 (0.04-0.32) | <0.001 | 0.32 (0.16-0.64) | 0.001 | 0.19 (0.07-0.48) | <0.001 |
| Age group |  |  |  |  |  |  |  |  |  |
| <15 years | 26 (43.3) | Reference | 0.060 | Reference | 0.075 | Reference | 0.096 | Reference | 0.161 |
| ≥15 years | 34 (56.7) | 1.67 (0.98-2.85) |  | 1.76 (0.95-3.28) |  | 1.55 (0.93-2.59) |  | 1.52 (0.85-2.74) |  |
| Sex | | | | | | | | | |
| Male | 37 (61.7) | Reference | 0.720 | Reference | 0.570 | Reference | 0.794 | Reference | 0.409 |
| Female | 23 (38.3) | 1.10 (0.64-1.90) |  | 1.20 (0.64-2.24) |  | 1.07 (0.64-1.81) |  | 1.29 (0.70-2.37) |  |
| Health seeking/Recruitment method | | | | | | | | | |
| Health Facility | 50 (83.3) | Reference | 0.111 | Reference | 0.620 | Reference | 0.199 | Reference | 0.497 |
| Community test and treat | 10 (16.7) | 1.81 (0.87-3.76) |  | 1.29 (0.48-3.47) |  | 1.57 (0.79-3.13) |  | 1.37 (0.55-3.41) |  |
| Presence of gametocytes at enrollment | | | | | | | | | |
| No | 13 (22.0) | Reference | 0.244 | Reference | 0.044 | Reference | 0.377 | Reference | 0.062 |
| Yes | 46 (78.0) | 0.68 (0.35-1.31) |  | 2.90 (1.03-8.18) |  | 0.75 (0.40-1.41) |  | 2.61 (0.95-7.12) |  |

Model e: Initial HRP2 concentration, adjusted for by: age, sex, health seeking*, initial parasite density, gametocyte at enrollment

|  | N=60*  n (%) | Standard RDT time to negativity | | | | uRDT time to negativity | | | |
| --- | --- | --- | --- | --- | --- | --- | --- | --- | --- |
|  |  | HR (95% CI) | p-value | aHR** (95% CI) | p-value | HR (95% CI) | p-value | aHR** (95% CI) | p-value |
| HRP2 concentration at enrollment log10[(pg/mL)] | | | | | | | | | |
| <5.45 | 20 (33.3) | Reference |  | Reference |  | Reference |  | Reference |  |
| 5.45-6.54 | 20 (33.3) | 0.45 (0.23-0.88) | 0.019 | 0.75 (0.33-1.70) | 0.494 | 0.45 (0.23-0.87) | 0.018 | 0.62 (0.27-1.39) | 0.245 |
| ≥6.54 | 20 (33.3) | 0.34 (0.17-0.68) | <0.001 | 0.62 (0.24-1.58) | 0.314 | 0.32 (0.16-0.63) | 0.001 | 0.42 (0.17-1.06) | 0.066 |
| Age group |  |  |  |  |  |  |  |  |  |
| <15 years | 26 (43.3) | Reference | 0.060 | Reference | 0.054 | Reference | 0.096 | Reference | 0.088 |
| ≥15 years | 34 (56.7) | 1.67 (0.98-2.85) |  | 1.90 (0.99-3.64) |  | 1.55 (0.93-2.59) |  | 1.70 (0.92-3.11) |  |
| Sex | | | | | | | | | |
| Male | 37 (61.7) | Reference | 0.720 | Reference | 0.419 | Reference | 0.794 | Reference | 0.270 |
| Female | 23 (38.3) | 1.10 (0.64-1.90) |  | 1.30 (0.68-2.49) |  | 1.07 (0.64-1.81) |  | 1.41 (0.77-2.61) |  |
| Health seeking/Recruitment method | | | | | | | | | |
| Health Facility | 50 (83.3) | Reference | 0.111 | Reference | 0.599 | Reference | 0.199 | Reference | 0.456 |
| Community test and treat | 10 (16.7) | 1.81 (0.87-3.76) |  | 1.31 (0.48-3.62) |  | 1.57 (0.79-3.13) |  | 1.43 (0.56-3.63) |  |
| Initial parasite density by qPCR, log10 (parasites/uL) | | | | | | | | | |
| < 2.93 | 19 (33.3) | Reference |  | Reference |  | Reference |  | Reference |  |
| 2.93-3.82 | 19 (33.3) | 0.60 (0.30-1.18) | 0.135 | 0.44 (0.16-1.17) | 0.098 | 0.76 (0.40-1.44) | 0.394 | 0.73 (0.29-1.86) | 0.514 |
| >3.83 | 19 (33.3) | 0.23 (0.11-0.49) | <0.001 | 0.17 (0.05-0.60) | 0.006 | 0.32 (0.16-0.64) | 0.001 | 0.34 (0.11-1.07) | 0.066 |
| Presence of gametocytes at enrollment | | | | | | | | | |
| No | 13 (22.0) | Reference | 0.244 | Reference | 0.076 | Reference | 0.377 | Reference | 0.097 |
| Yes | 46 (78.0) | 0.68 (0.35-1.31) |  | 2.64 (0.90-7.72) |  | 0.75 (0.40-1.41) |  | 2.40 (0.95-6.76) |  |

Model f: Gametocyte at enrollment, adjusted for by: age, sex, and health seeking*

|  | N=60*  n (%) | Standard RDT time to negativity | | | | uRDT time to negativity | | | |
| --- | --- | --- | --- | --- | --- | --- | --- | --- | --- |
|  |  | HR (95% CI) | p-value | aHR** (95% CI) | p-value | HR (95% CI) | p-value | aHR** (95% CI) | p-value |
| Presence of gametocytes at enrollment | | | | | | | | | |
| No | 13 (22.0) | Reference | 0.244 | Reference | 0.111 | Reference | 0.377 | Reference | 0.107 |
| Yes | 46 (78.0) | 0.68 (0.35-1.31) |  | 2.34 (0.82-6.66) |  | 0.75 (0.40-1.41) |  | 2.28 (0.84-6.21) |  |
| Age group |  |  |  |  |  |  |  |  |  |
| <15 years | 26 (43.3) | Reference | 0.060 | Reference | 0.033 | Reference | 0.096 | Reference | 0.042 |
| ≥15 years | 34 (56.7) | 1.67 (0.98-2.85) |  | 1.99 (1.06-3.76) |  | 1.55 (0.93-2.59) |  | 1.85 (1.02-3.35) |  |
| Sex | | | | | | | | | |
| Male | 37 (61.7) | Reference | 0.720 | Reference | 0.661 | Reference | 0.794 | Reference | 0.411 |
| Female | 23 (38.3) | 1.10 (0.64-1.90) |  | 1.16 (0.60-2.21) |  | 1.07 (0.64-1.81) |  | 1.29 (0.70-2.39) |  |
| Recruitment method | | | | | | | | | |
| Health Facility | 50 (83.3) | Reference | 0.111 | Reference | 0.956 | Reference | 0.199 | Reference | 0.813 |
| Community test and treat | 10 (16.7) | 1.81 (0.87-3.76) |  | 0.97 (0.33-2.81) |  | 1.57 (0.79-3.13) |  | 1.12 (0.43-2.92) |  |

Model g: DNA clearance rate, adjusted by: age, sex, health seeking*, initial parasite density

|  | N=60*  n (%) | Standard RDT time to negativity | | | | uRDT time to negativity | | | |
| --- | --- | --- | --- | --- | --- | --- | --- | --- | --- |
|  |  | HR (95% CI) | p-value | aHR** (95% CI) | p-value | HR (95% CI) | p-value | aHR** (95% CI) | p-value |
| DNA clearance rate, assessed by persistence of qPCR parasitemia (days) | | | | | | | | | |
| <7 | 28 (46.7) | Reference | <0.001 | Reference | 0.005 | Reference | <0.001 | Reference | 0.005 |
| ≥7 | 32 (53.3) | 0.30 (0.16-0.56) |  | 0.39 (0.20-0.76) |  | 0.34 (0.19-0.61) |  | 0.40 (0.21-0.76) |  |
| Age group |  |  |  |  |  |  |  |  |  |
| <15 years | 26 (43.3) | Reference | 0.060 | Reference | 0.260 | Reference | 0.096 | Reference | 0.047 |
| ≥15 years | 34 (56.7) | 1.67 (0.98-2.85) |  | 2.00 (1.09-3.67) |  | 1.55 (0.93-2.59) |  | 1.79 (1.01-3.17) |  |
| Sex | | | | | | | | | |
| Male | 37 (61.7) | Reference | 0.720 | Reference | 0.668 | Reference | 0.794 | Reference | 0.648 |
| Female | 23 (38.3) | 1.10 (0.64-1.90) |  | 1.15 (0.61-2.15) |  | 1.07 (0.64-1.81) |  | 1.15 (0.63-2.10) |  |
| Health seeking/Recruitment method | | | | | | | | | |
| Health Facility | 50 (83.3) | Reference | 0.111 | Reference | 0.423 | Reference | 0.199 | Reference | 0.610 |
| Community test and treat | 10 (16.7) | 1.81 (0.87-3.76) |  | 0.68 (0.26-1.76) |  | 1.57 (0.79-3.13) |  | 0.80 (0.34-1.89) |  |
| Initial parasite density by qPCR, log10 (parasites/uL) | | | | | | | | | |
| < 2.93 | < 2.93 | Reference |  | Reference |  | Reference |  | Reference |  |
| 2.93-3.82 | 2.93-3.82 | 0.60 (0.30-1.18) | 0.135 | 0.57 (0.24-1.34) | 0.199 | 0.76 (0.40-1.44) | 0.394 | 0.85 (0.40-1.85) | 0.687 |
| >3.83 | >3.83 | 0.23 (0.11-0.49) | <0.001 | 0.24 (0.09-0.63) | 0.004 | 0.32 (0.16-0.64) | 0.001 | 0.42 (0.17-1.00) | 0.049 |

Supplemental figure 2. Kaplan Meier curves of proportion remaining RDT or uRDT positive by days since treatment, stratified by age category, n=137 participants.


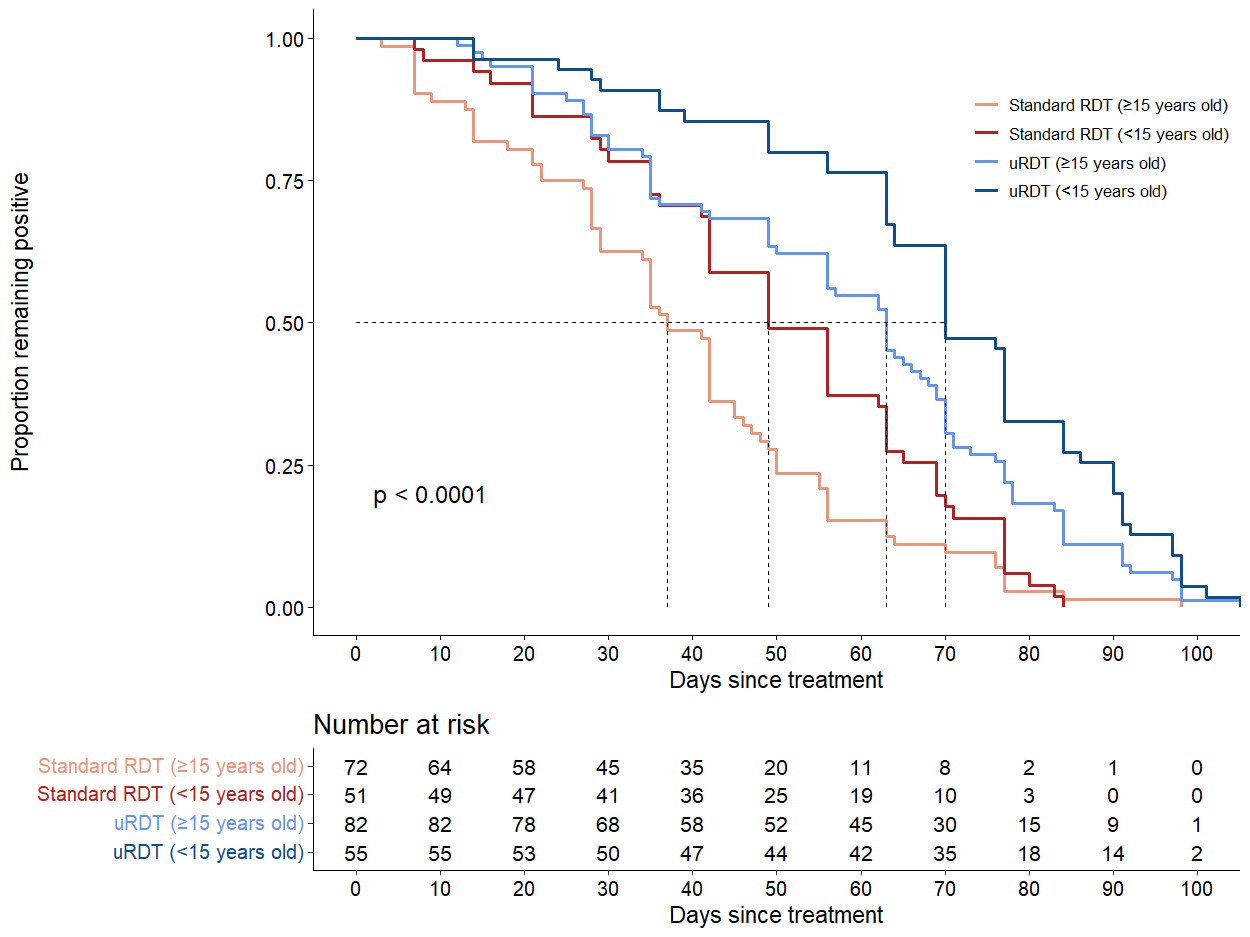


Supplemental figure 3. Kaplan Meier curves of proportion remaining RDT or uRDT positive by days since treatment, stratified by HRP concentration (log10 pg/mL), n=137 participants.


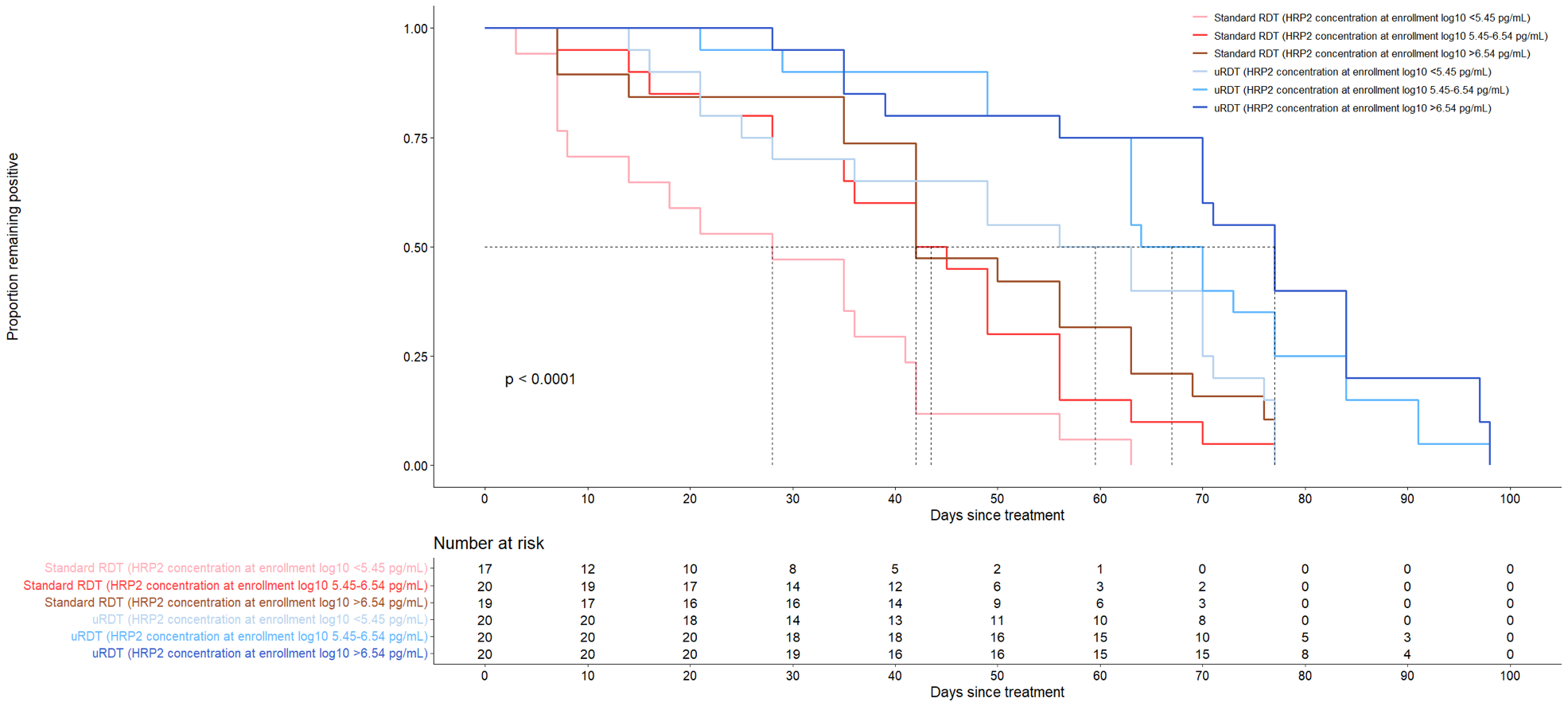


Supplemental figure 4. Haplotypes of longitudinal samples among 3 participants that had uRDT positivity beyond the median duration of 67 days.


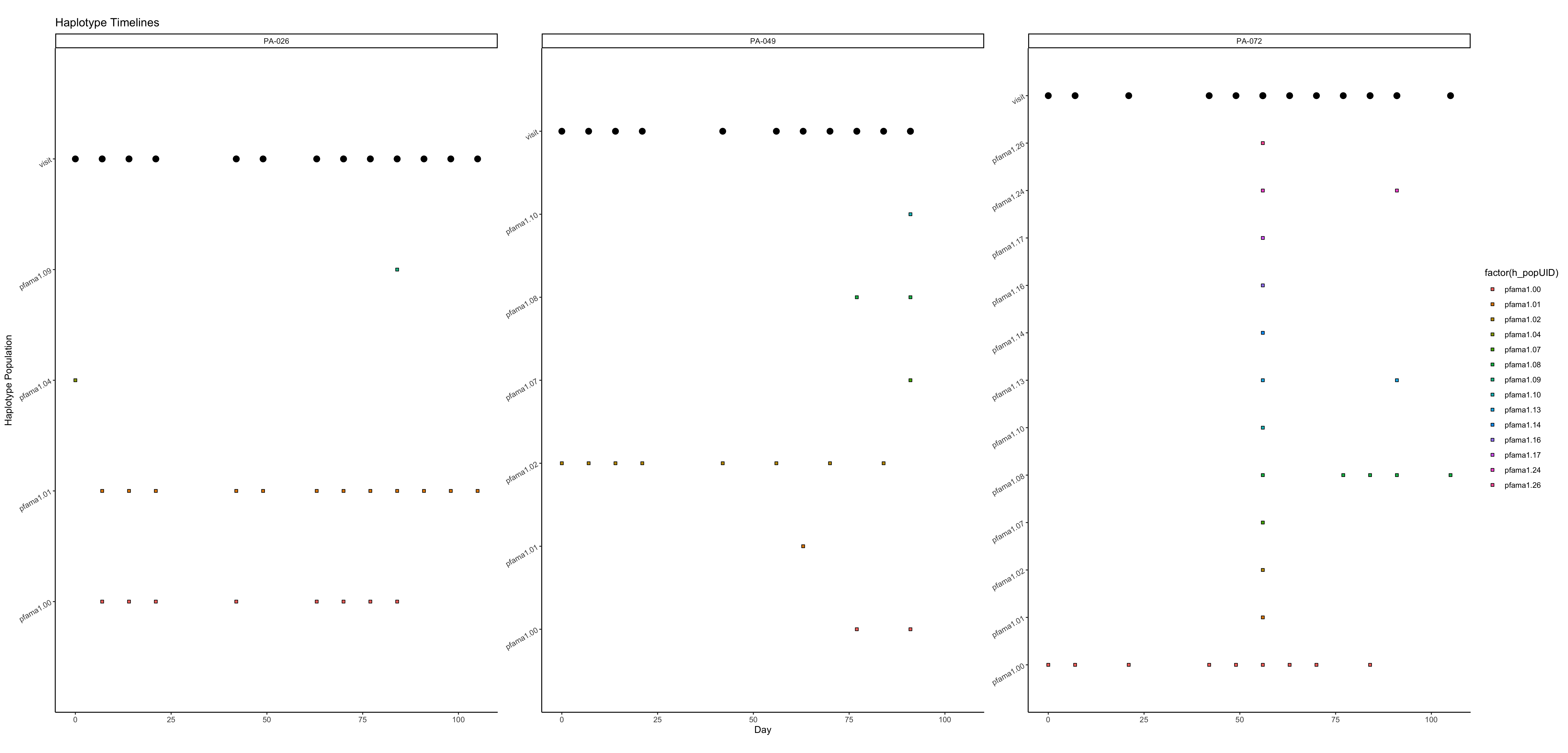


Supplemental Tables 3-5. Treatment efficacy study (TES) sub-analysis

The TES of artemether-lumefantrine (AL) was conducted as a secondary analysis. Inclusion criteria for a patient to enter the sub-study were based on WHO guidelines for the Treatment of Malaria (3rd ed. Geneva, Switzerland, 2015): febrile patients of all ages with uncomplicated malaria evidenced by RDT criteria with no history of antimalarial drug use in the prior two weeks. Exclusion criteria included pregnancy, mixed infections, breastfeeding, or malnutrition [1]. Additional exclusion criteria not having Day 0 blood sample to confirm infection status / parasitemia, censored incomplete lab results, withdrawal, and participants lost in follow up. WHO recommends for a TES in a low-transmission setting a sample size of N=50 [1]. After applying exclusion criteria, our sample size was N=44. The first dose of AL and single dose of primaquine were observed in the clinic, further doses of AL were self-administered. Patients were then followed weekly until they tested negative by RDT and uRDT for two weeks in a row. At each follow-up visit, participants were asked about adverse events and severe malaria symptoms. Participants were also encouraged to call the local study nurse at a toll-free number if any problems arise, ensuring safety was assessed both actively and passively.

At each follow-up visit, we assessed for early treatment failure, late clinical failure, late parasitological failure, and adequate clinical and parasitological response based on the WHO guidelines. Early treatment failure (ETF) is classified as the development of danger signs or severe malaria on days 1, 2, or 3 in the presence of parasitemia. Late clinical failure (LCF) is classified as the development of danger signs or severe malaria after day 3 or the presence of parasitemia and axillary temperature ≥ 37.5 °C on any day from Day 4 to Day 28, without previously meeting any of the criteria of ETF. Late parasitological failure (LPF) is classified as the presence of parasitemia on any day from Day 7 to Day 28 and axillary temperature under 37.5 °C, without previously meeting any of the criteria for ETF or LCF. Adequate clinical and parasitological response (ACPR) is classified as the absence of parasitemia on day 28 irrespective of axillary temperature without previously meeting any of the criteria for ETC, LCF, or LPF. As microscopy was not conducted, we defined parasite clearance as parasite density <200 parasites/µL of blood which is the accepted minimum level of sensitivity of microscopy.

Our analysis group (N=44 patients) included febrile malaria patients detected at the health facility on the first day of treatment (day 0). Out of the 164 individuals originally enrolled in the longitudinal cohort study, participants were excluded that were lost to follow-up, enrolled after Day 0, enrolled through mass screening and treatment, and had incomplete lab results. Baseline characteristics of those that were included in the TES analysis are shown in Supplemental Table 3 below. 59% of participants were male (n=26) and 45 were above age 15 years old (n=20).

AL efficacy results are displayed in Supplemental Tables 4 and 5 below. At Day 7 (the first time point with parasite density data), 100% of patients had successfully cleared the infection. This clearance success was maintained at days 14, 21, 28, and 42. At Day 35, two patients were afebrile and had a low parasite density infection of 550 and 493 and parasites/µL respectively, but at Day 42 both patients were clear again. At Day 7 and 28, 1 patient presented with a fever (≥37.5°C) but they were PCR-negative, suggesting their fever was not due to malaria. In both active and passive pharmacovigilance, no patients reported adverse events during the follow-up period. In a sub-analysis of the 10 participants that had a Day 0 qPCR parasite density >1000 parasites/µL, there were no differences in any of the study outcomes.

Supplemental Table 3. TES participant baseline characteristics


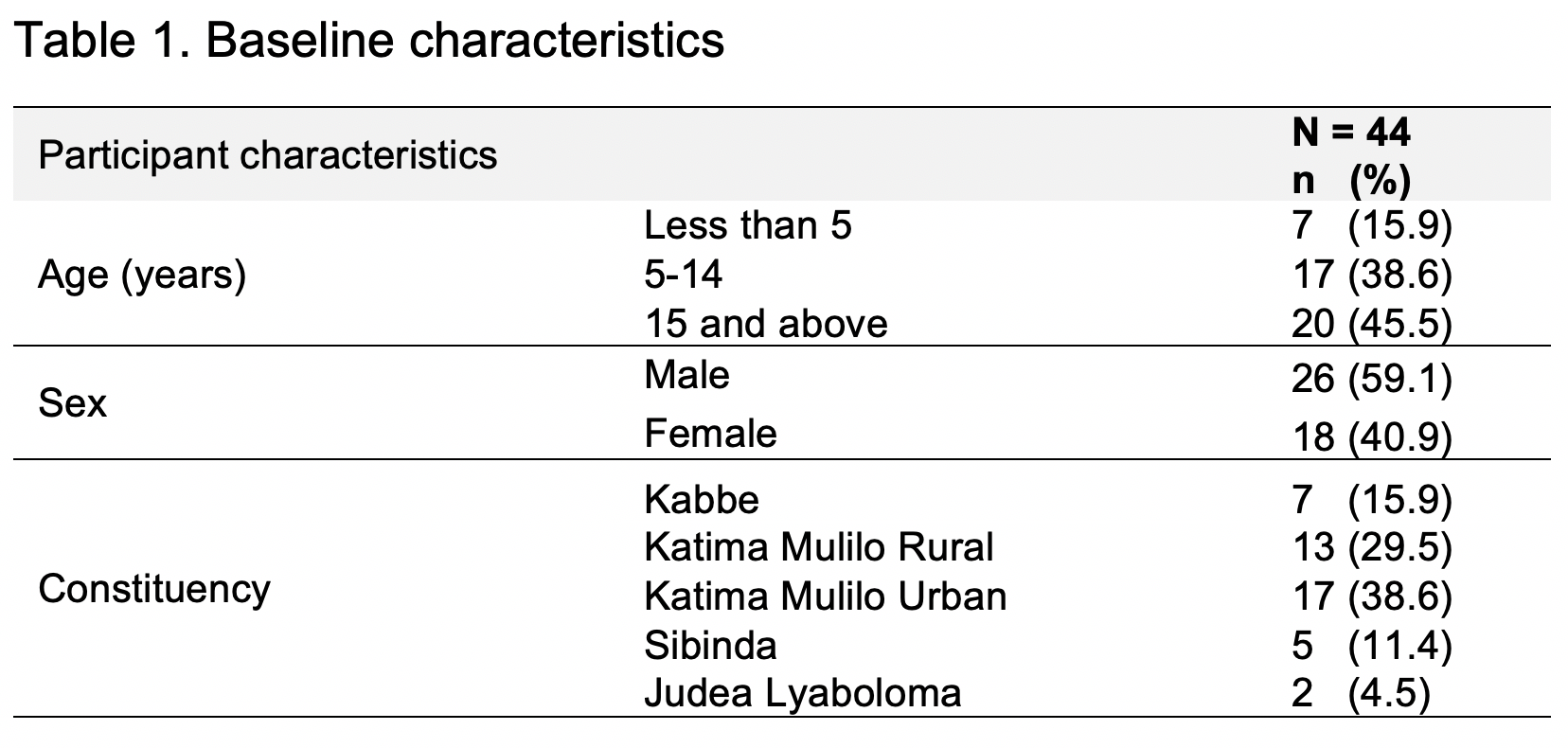


Supplemental Table 4. Infection clearance and fever at follow-up visits

|  | Infection cleared  N=44  n (%) | Fever at follow-up visit (≥37.5°C)  n (%) |
| --- | --- | --- |
| Day 7 | 44 (100%) | 1 (2%) |
| Day 14 | 44 (100%) | 0 (0%) |
| Day 21 | 44 (100%) | 0 (0%) |
| Day 28 | 44 (100%) | 1 (2%) |
| Day 35 | 42 (95%) | 0 (0%) |
| Day 42 | 44 (100%) | 0 (0%) |

Supplemental Table 5. TES outcomes

|  | Infection cleared  N=44  n (%) |
| --- | --- |
| ETF | 0 (0%) |
| LCF | 2 (5%) |
| LPF | 0 (0%) |
| ACPR | 42 (95%) |

ETF: early treatment failure; LCF: late clinical failure; LPF: late parasitological failure; ACPR: adequate clinical and parasitological response
